# Brain metabolic connectivity in ALS due to *C9ORF72* hexanucleotide expansion: a [^18^F]FDG-PET study

**DOI:** 10.1007/s00259-025-07705-1

**Published:** 2025-12-11

**Authors:** Antonio Canosa, Stefano Callegaro, Umberto Manera, Rosario Vasta, Sara Cabras, Francesca Di Pede, Filippo De Mattei, Francesca Palumbo, Barbara Iazzolino, Anastasia Dei Giudici, Enrico Matteoni, Grazia Zocco, Emilio Minerva, Alessandra Maccabeo, Giorgio Pellegrino, Daniela Pascariu, Maurizio Grassano, Pietro Piombino, Marcella Testa, Giulia Polverari, Giuseppe Fuda, Ilaria Merulla, Federico Casale, Salvatore Gallone, Cristina Moglia, Andrea Calvo, Marco Pagani, Adriano Chiò

**Affiliations:** 1 ALS Centre, ‘Rita Levi Montalcini’ Department of Neuroscience, University of Turin, Via Cherasco 15, Turin, 10126 Italy; 2 Neurology Unit 1U, Azienda Ospedaliero-Universitaria Città della Salute e della Scienza di Torino, Turin, Italy; 3 Institute of Cognitive Sciences and Technologies, C.N.R., Rome, Italy; 4 Neuroscience Institute of Turin (NIT), Turin, Italy; 5 Centre for Neuroscience, University of Camerino, Camerino, Italy; 6 Positron Emission Tomography Centre AFFIDEA-IRMET S.p.A, Turin, Italy; 7 Department of Medical Radiation Physics and Nuclear Medicine, Karolinska University Hospital, Stockholm, Sweden

**Keywords:** Amyotrophic lateral sclerosis, *C9ORF72*, [^18^F]FDG-PET, Connectivity

## Abstract

**Purpose:**

Our aim was to investigate brain metabolic connectivity, as assessed via [^18^F]FDG-PET, in ALS patients carrying the *C9ORF72* expansion (*C9*-ALS).

**Methods:**

We compared brain metabolism of *C9*-ALS and patients without mutations of the main ALS-related genes (ctrl-ALS) through the two-sample t-test model of SPM12. Metabolic clusters showing a significant difference between the two groups were used as seed regions for an interregional correlation analysis (IRCA) in each group to evaluate metabolic connectivity.

**Results:**

As compared to ctrl-ALS, *C9*-ALS showed a relative hypometabolism in bilateral thalamus and left precentral and postcentral gyri, and a relative hypermetabolism in bilateral cerebellum and brainstem. In the IRCA, a positive correlation was found between the thalamic seed region and the cingulate cortex, including its anterior part. This correlation was broader in *C9*-ALS than in Ctrl-ALS. A negative correlation between the thalamic seed region and the sensorimotor cortex was only found in *C9*-ALS. In the IRCA, based on the cerebellar/brainstem cluster, positive correlations with the seed region substantially represented autocorrelation in both groups. Negative correlation, which mainly included frontal cortices, was more extensive in *C9*-ALS than in Ctrl-ALS.

**Conclusion:**

In the comparison with ctrl-ALS, *C9*-ALS showed a relatively lower metabolism in the thalami and a relatively higher metabolism in the brainstem and the cerebellum. As compared to ctrl-ALS, *C9*-ALS showed a predominant involvement of the salience network, which is related to cognitive and behavioural control. The cerebellum might be recruited to cope with cognitive impairment to a greater extent in *C9*-ALS than in ctrl-ALS.

## Introduction

Amyotrophic lateral sclerosis (ALS) is a heterogeneous neurodegenerative disease affecting both motor and extramotor systems, with approximately 50% of patients displaying cognitive and/or behavioural deficits that fall within the spectrum of frontotemporal dementia (FTD) [1]. Overall, approximately 10% of ALS patients have a positive family history for ALS and/or FTD. The *C9ORF72* expansion is the most common cause of genetic ALS [2] and is a strong risk factor for cognitive impairment up to full-blown FTD in these patients [3]. Frontotemporal syndromes associated with ALS are typically characterised by deficits in executive function, language, and behaviour [4]. In addition, FTD due to *C9ORF72* mutation has peculiar manifestations. Late-onset psychosis and bipolar disorder can be the initial prodromal phase of dementia [5]. Furthermore, a recent study pointed out that a previous psychopathological history, including depressive disorder, post-traumatic stress disorder and substance abuse or dependence, is more frequently detected in *C9ORF72* expansion carriers compared to the general population [6]. Neuroimaging studies on *C9ORF72* expansion carriers consistently support the central role of this gene in the ALS-FTD continuum. Brain Magnetic Resonance Imaging (MRI) studies show that symptomatic *C9ORF72* carriers have increasing ventricular volumes and decreasing bilateral thalamic volumes over time, compared to both control and sporadic ALS groups. These changes are accompanied by prominent cortical thinning in bilateral frontal and temporal regions [7]. On the other hand, 2-[^18^F]fluoro-2-deoxy-D-glucose-Positron-Emission Tomography ([^18^F]FDG-PET) imaging reveals relative hypometabolism in the anterior/posterior cingulate cortex, insula, caudate, thalamus, frontal cortex, and superior temporal lobe, as well as relative hypermetabolism in the midbrain, occipital cortex, globus pallidus, and middle/inferior temporal regions in ALS patients carrying the *C9ORF72* expansion compared to non-mutated patients [8]. With regard to the investigation of brain connectivity, an MRI-based study showed that *C9ORF72* ALS patients have a greater involvement of frontal white matter tracts and enhanced functional connectivity of the visual network than ALS patients not carrying the expansion [9].

This study aimed to investigate brain metabolic connectivity in *C9ORF72* patients by applying [^18^F]FDG-PET to highlight differences compared with patients without the mutation.

## Materials and methods

### Participants

We included 70 patients carrying the *C9ORF72* expansion (*C9*-ALS), diagnosed with genetically determined ALS according to El Escorial revised diagnostic criteria [10] at the ALS Centre of Turin (‘Rita Levi Montalcini’ Department of Neuroscience, University of Turin, Turin, Italy), between 2008 and 2022. A comparison cohort of 70 patients diagnosed with definite, probable, and probable laboratory-supported ALS according to El Escorial revised diagnostic criteria [10] was randomly collected from the series of 891 subjects who underwent brain [^18^F]FDG-PET at diagnosis in the same time period at the ALS Centre of Turin. These subjects did not carry any mutations in the major ALS-related genes (i.e. *SOD1*, *TARDBP*, *FUS*, *C9ORF72*) and were labeled as control ALS (ctrl-ALS). To ensure the representativeness of both the *C9-* and the ctrl-ALS samples, we systematically compared their principal demographic and clinical variables (i.e., age at onset, sex, cognitive status according to international diagnostic criteria [11], diagnostic delay, and ΔALSFRS-R) against those of the corresponding register population (Piemonte and Valle d’Aosta Register, PARALS [12]) up to 2022. The diagnostic delay was calculated as the time interval in months between the onset of symptoms and the date of diagnosis. The ΔALSFRS-R was calculated as the number of points lost per month from disease onset to diagnosis. These comparisons, as well as the direct comparison between *C9*-ALS and ctrl-ALS were based on the following tests: the Chi-square test for nominal categorical variables; the non-parametric Kruskal-Wallis test for continuous quantitative variables with significant Levene test and Shapiro-Wilk test results, where the assumptions of homogeneity of variances and normality of distribution required for ANOVA were not met.

### Genetic analysis and neuropsychological assessment

We reported the procedure for genetic screening and neuropsychological evaluation elsewhere [3, 13].

### [^18^F]FDG-PET image acquisition and pre-processing

Brain [^18^F]FDG-PET was performed according to published guidelines [14]. Patients fasted at least 6 h before the examination. Blood glucose was < 7.2 mmol/l in all cases before the procedure. After a 20-min rest, 185–200 MBq of [^18^F]FDG was injected. The acquisition started 60 min after the injection. PET/CT scans were performed on a Discovery ST-E System (General Electric). Acquisition duration was approximately 15 min. Brain CT and PET scans were performed sequentially. The former was used for attenuation correction of the PET data. The PET images were reconstructed with four iterations and 28 subsets with an initial voxel size of 2.34 × 2.34 × 2.00 mm, and data were collected in 128 × 128 matrices. The reconstruction algorithm was VUE Point HD. It does not include PSF and TOF corrections. The number of slices was 47. Images were spatially normalised to a customised brain [^18^F]FDG-PET template [15] and subsequently smoothed with a 10-mm filter in MATLAB R2018b (MathWorks, Natick, MA, USA). The voxel size after spatial normalisation into the standard brain space is 2 × 2 × 2 mm. Intensity normalisation was performed at individual level by dividing the value at each voxel by the mean value of the whole brain.

### Statistical analysis of [^18^F]FDG-PET data

We compared *C9*-ALS and ctrl-ALS through the two-sample t-test model of SPM12. We did not include any covariates in this analysis after proving that the *C9*-ALS and the ctrl-ALS samples were adequately representative of the patients carrying and not carrying the *C9ORF72* expansion in the ALS population of the PARALS. Therefore, any differences in demographic and clinical features between the two samples (*C9*-ALS and the ctrl-ALS) are expected reflect phenotypic variations between these two subgroups of patients within the ALS population. In this comparison the height threshold was set at *P* < 0.001 (*P* < 0.05 FWE-corrected at cluster level). Metabolic clusters showing a significant difference between *C9*-ALS and ctrl-ALS were then used as seed regions in a multiple regression analysis in each patient group to identify cerebral regions whose metabolism was positively or negatively correlated with that of the seed clusters (i.e., interregional correlation analysis, IRCA). IRCA applied to [^18^F]FDG-PET is a statistical method used to assess metabolic connectivity in the brain by evaluating the correlation of glucose metabolism between different brain regions. Regions showing high interregional correlation are interpreted as being metabolically connected. This method provides insights into the network organization of the brain. In this method, the [^18^F]FDG uptake values from regions of interest (*seed regions*) are extracted, and their correlation with other brain regions is computed to assess the degree of metabolic coupling between brain areas. The resulting correlation patterns reflect large-scale network organisation and can reveal alterations in functional integration associated with neurological disorders. For the IRCA we set the height threshold at *P* < 0.001 as well (*P* < 0.05 FWE-corrected at cluster level). In all the analyses only clusters containing > 125 contiguous voxels were considered significant. Brodmann areas (BAs) were identified at a 0–2-mm range from the Talairach coordinates of the SPM output isocenters corrected by Talairach Client (http://www.talairach.org/index.html).

### Compliance with reporting guidelines

We have followed the STROBE reporting guideline.

## Results

### Demographic and clinical features of participants

The demographic and clinical characteristics of *C9*-ALS and ctrl-ALS included in the study are reported in Table 1, in comparison with the PARALS population. The ctrl-ALS group showed no statistically significant differences compared to the subjects with normal genetic screening from the register population of the PARALS in any of the considered variables. By contrast, the *C9*-ALS group differed from the *C9ORF72* positive subjects of the register population only in terms of Strong category distribution, showing a statistically significant lower proportion of patients with comorbid frontotemporal dementia (ALS/FTD). In the direct comparison between *C9*-ALS and ctrl-ALS included in the study, *C9*-ALS patients showed a significantly lower age at onset (*p* = 0.0005). No other significant difference was found for the variables reported in Table 1.

**Table 1:** Demographic and clinical characteristics of *C9*-ALS and ctrl-ALS: comparison of each group with the corresponding subjects of the Piemonte and Valle d’Aosta Register for ALS (PARALS) population

	*C9-*ALS (study sample) (*n* = 70)	*C9-*ALS (PARALS) (*n* = 143)	*p*-value
Sex, male [n(%)]	37 (53)	72 (50)	0.843
Cognitive classification, [n(%)]			0.017
∙ ALSbi	7 (10)	4 (3)	
∙ ALSci	11 (16)	21 (15)	
∙ ALScbi	1 (1)	4 (3)	
∙ CN	28 (40)	34 (23)	
∙ FTD	10 (14)	40 (28)	
∙ Missing	13 (19)	40 (28)	
Age at onset, years [median (IQR)]	57.8 (50.7 – 64.4)	61.3 (53.9 – 67.1)	0.051
Diagnostic delay, years [median (IQR)]	0.8 (0.5 – 1.0)	0.7 (0.4 – 1.1)	0.372
ΔALSFRS-R, points/months [median (IQR)]	0.5 (0.3 – 0.9)	0.7 (0.4 – 1.1)	0.064
	Ctrl-ALS (study sample) (*n* = 70)	Ctrl-ALS (PARALS) (*n* = 1808)	*p*-value
Sex, male [n(%)]	39 (56)	1004 (56)	1.000
Cognitive classification, [n(%)]			0.188
∙ALSbi	5 (7)	90 (5)	
∙ALSci	5 (7)	212 (12)	
∙ALScbi	2 (3)	72 (4)	
∙CN	33 (48)	559 (31)	
∙FTD	4 (5)	150 (8)	
∙Missing	21 (30)	725 (40)	
Age at onset, years [median (IQR)]	66.2 (55.7 – 73.0)	67.3 (59.3 – 73.8)	0.211
Diagnostic delay, years [median (IQR)]	0.8 (0.6 – 1.2)	0.8 (0.4 – 1.2)	0.246
ΔALSFRS-R, points/months [median (IQR)]	0.6 (0.4 – 1.1)	0.6 (0.3 – 1.3)	0.846

Data were compared using Mann Whitney U test for continuous variables and chi-square test for categorical variables

ALSbi: ALS with behavioural impairment. ALSci: ALS with cognitive impairment. ALScbi: ALS with cognitive and behavioural impairment. CN: cognitively normal. ALS-FTD: ALS with frontotemporal dementia

### [^18^F]FDG-PET data: direct comparison between *C9*-ALS and ctrl-ALS

To address the soundness of the whole brain as a normalisation factor, we have followed the procedure by Borghammer et al. [16]. Specifically, we have extracted the global whole brain mean metabolic values for both *C9*-ALS and ctrl-ALS and performed a two-sample t-test to address whether the normalisation factors are statistically different or not between the two groups, possibly leading to biased normalisation. The average (+/- SD) metabolic intake for *C9*-ALS is 1588.43 +/- 375.76, compared to 1560.54 +/- 381.90 for ctrl-ALS (+ 1.8% difference of *C9*-ALS *versus* ctrl-ALS). The two-sample t-test yielded *p* = 0.672 (t = 0.4243, CI at 95% = [-102.14, 157.92]), not rejecting the null hypothesis that the two groups have equal means, suggesting the whole brain as a sound normalisation factor between the two groups (as there are no metabolic a-priori differences that would undermine the normalisation).

**Fig. 1:**
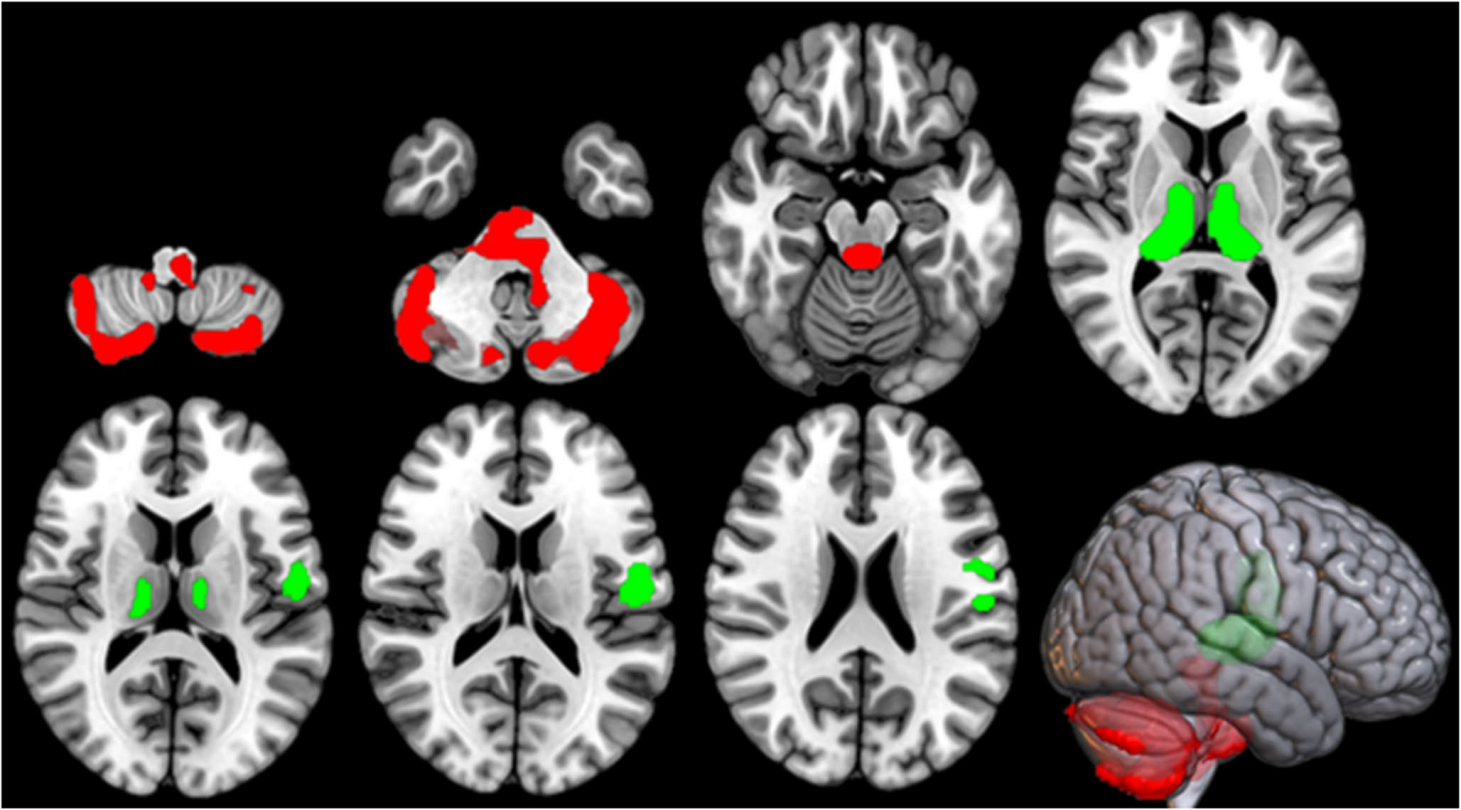
The regions showing a statistically significant relative hypometabolism in *C9*-ALS patients as compared to ctrl-ALS subjects are marked in green, while the regions showing a statistically significant relative hypermetabolism in *C9*-ALS patients as compared to ctrl-ALS subjects are marked in red. The clusters are reported on axial sections of a brain Magnetic Resonance Imaging template and on the brain surface of a glass brain rendering (bottom right)

*C9*-ALS showed clusters of relative hypometabolism in bilateral thalamus (bilateral pulvinar and left medial dorsal nucleus) and left precentral and postcentral gyri, and clusters of relative hypermetabolism in bilateral cerebellum and brainstem (Fig. 1; Table 2). As seed regions for the IRCA, we focused on the thalamic cluster and the cerebellar/brainstem cluster, based on the peculiar involvement of these regions in *C9ORF72*-related ALS [8, 17]. No further segmentation of the seed regions was performed according to side or subregions. In addition, we have also run the analyses by adding age at PET as covariate, since this variable represented the only significant difference between the two study groups among demographic and clinical features. The clusters of relative hypo- and hypermetabolism of *C9*-ALS compared to ctrl-ALS found in the main analyses were still present after the correction for age at PET (data not shown).

**Table 2:** Clusters of relative hypometabolism and hypermetabolism of *C9*-ALS compared to ctrl-ALS. BA: Brodmann area

Clusters of relative hypometabolism of *C9-*ALS compared to ctrl-ALS							
*P (FWE- corrected)*	*Cluster extent*	*Z-score*	*Talairach coordinates (x*,* y*,* z)*			*Brain region*	*Sub-region*
0.000	2,005	6.84	20	-29	5	Right Thalamus	Pulvinar
		6.45	-16	-29	3	Left Thalamus	Pulvinar
		6.19	-6	-13	4	Left Thalamus	Medial Dorsal Nucleus
0.011	794	4.32	-51	-13	15	Left Postcentral Gyrus	BA 43
		4.07	-59	-8	34	Left Precentral Gyrus	BA 6
Clusters of relative hypermetabolism of *C9-*ALS compared to ctrl-ALS							
*P (FWE- corrected)*	*Cluster extent*	*Z-score*	*Talairach coordinates (x*,* y*,* z)*			*Brain region*	*Sub-region*
0.000	2,441	6.61	0	-35	-5	Left Cerebellar Anterior Lobe	Culmen
		4.47	-6	-37	-37	Left Cerebellar Posterior Lobe	Cerebellar Tonsil
		4.07	14	-30	-24	Right Cerebellar Anterior Lobe	Culmen
		3.44	12	-39	-38	Right Cerebellar Posterior Lobe	Cerebellar Tonsil
0.000	6,129	4.71	-40	-52	-33	Left Cerebellar Posterior Lobe	Cerebellar Tonsil
		4.41	-12	-77	-21	Left Cerebellar Posterior Lobe	Declive
		4.28	42	-49	-36	Right Cerebellar Posterior Lobe	Cerebellar Tonsil
		4.25	-34	-81	-31	Left Cerebellar Posterior Lobe	Pyramis
		4.19	36	-65	-22	Right Cerebellar Posterior Lobe	Declive
		4.18	14	-71	-47	Right Cerebellar Posterior Lobe	Inferior Semi-Lunar Lobule
		4.07	-12	-69	-47	Left Cerebellar Posterior Lobe	Inferior Semi-Lunar Lobule
		3.70	50	-70	-30	Right Cerebellar Posterior Lobe	Tuber
		3.70	50	-66	-32	Right Cerebellar Posterior Lobe	Pyramis

### [^18^F]FDG-PET data: IRCA in *C9*-ALS

**Fig. 2:**
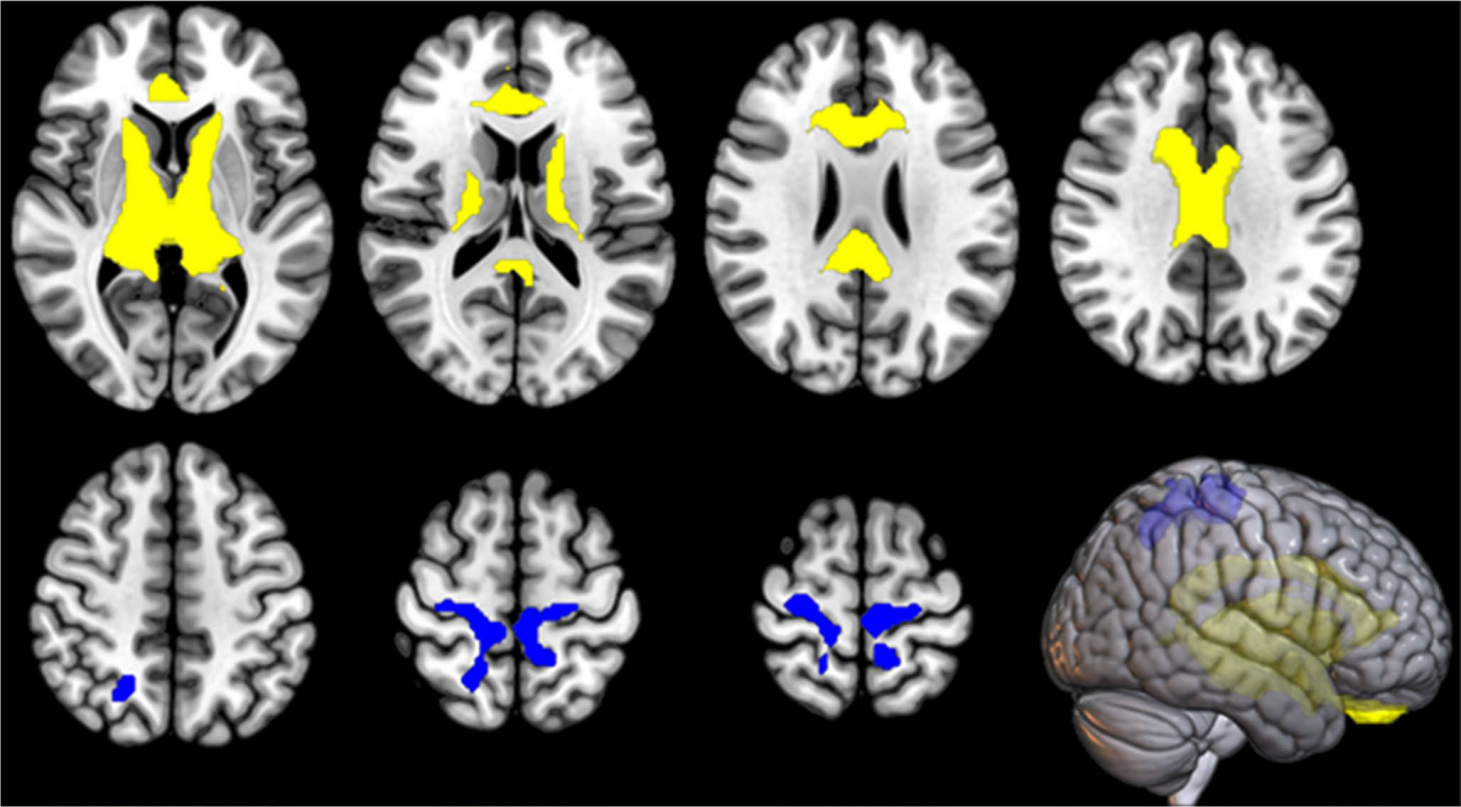
IRCA in *C9*-ALS: clusters of positive (marked in yellow) and negative (marked in blue) correlation with the thalamic cluster (seed region) are reported on axial sections of a brain Magnetic Resonance Imaging template and on the brain surface of a glass brain rendering (bottom right)

In the IRCA in *C9*-ALS patients, we found that the thalamic cluster metabolism was positively correlated with the metabolism of large bilateral thalamic regions, bilateral cingulum and right caudate, and negatively correlated with the metabolism of right precuneus, precentral and postcentral gyri, and left medial frontal gyrus and paracentral lobule (Fig. 2; Table 3).

**Table 3:** IRCA in *C9*-ALS: clusters of positive and negative correlation with the thalamic cluster (seed region). BA: Brodmann area

IRCA in *C9*-ALS: cluster of positive correlation with the thalamic cluster							
*P (FWE- corrected)*	*Cluster extent*	*Z-score*	*Talairach coordinates (x*,* y*,* z)*			*Brain region*	*Sub-region*
0.000	12,849	65,535	10	-9	6	Right Thalamus	Ventral Lateral Nucleus
		65,535	-6	-10	4	Left Thalamus	*
		65,535	12	-23	5	Right Thalamus	*
		6.12	16	15	27	Right Cingulate Gyrus	BA 32
		5.94	4	-6	28	Right Cingulate Gyrus	BA 24
		5.93	4	-12	28	Right Cingulate Gyrus	BA 23
		5.79	-6	7	27	Left Cingulate Gyrus	BA 24
		5.78	8	9	25	Right Anterior Cingulate	BA 33
		5.55	16	12	9	Right Caudate	Caudate Body
		5.50	-4	15	23	Left Anterior Cingulate	BA 24
IRCA in *C9*-ALS: cluster of negative correlation with the thalamic cluster							
*P (FWE- corrected)*	*Cluster extent*	*Z-score*	*Talairach coordinates (x*,* y*,* z)*			*Brain region*	*Sub-region*
0.000	1,437	4.15	20	-52	52	Right Precuneus	BA 7
		3.87	16	-28	55	Right Sub-Gyral	BA 4
		3.81	-10	-22	62	Left Medial Frontal Gyrus	BA 6
		3.73	28	-18	62	Right Precentral Gyrus	BA 6
		3.69	-8	-40	59	Left Paracentral Lobule	BA 5
		3.23	18	-45	67	Right Postcentral Gyrus	BA 7

**Fig. 3:**
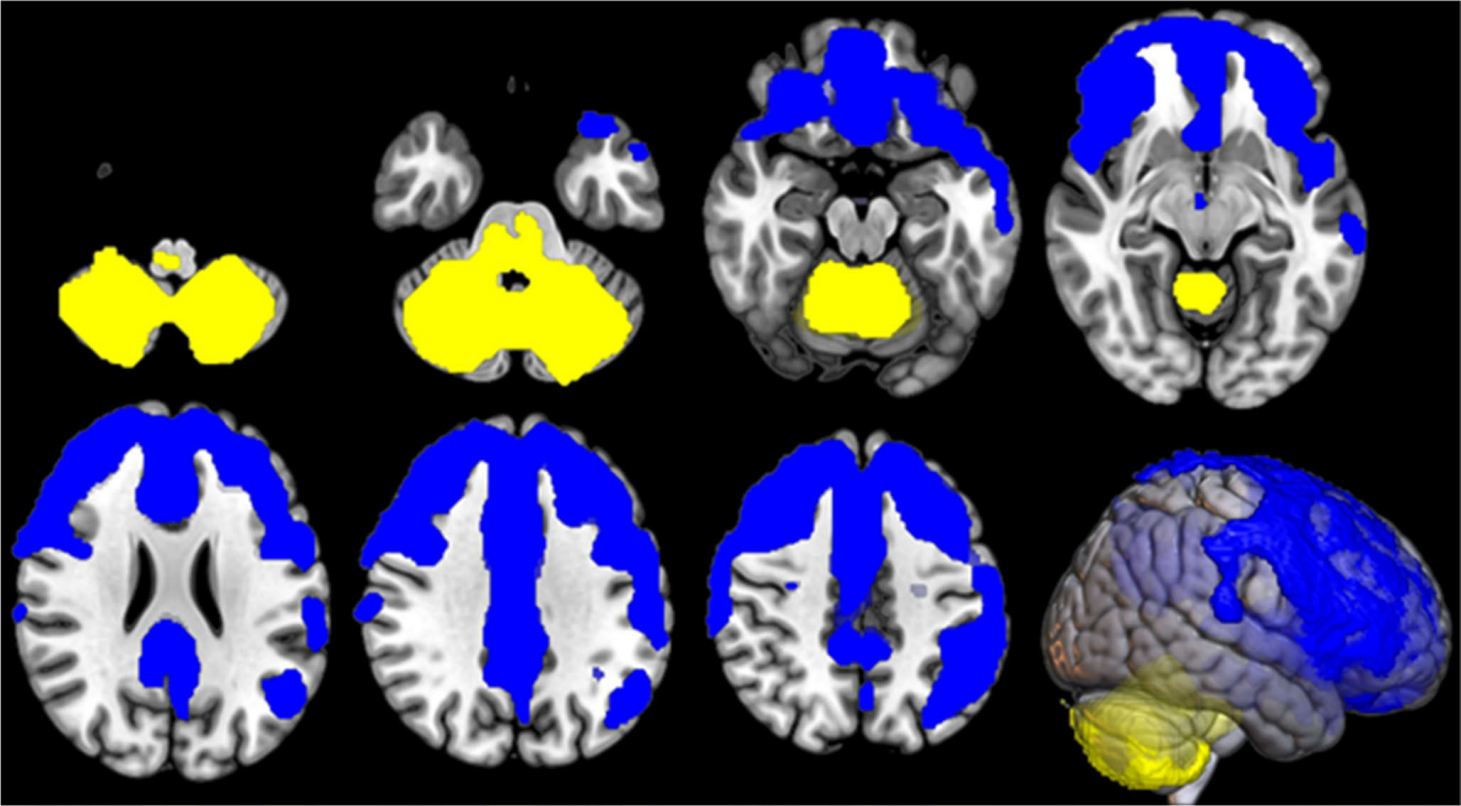
IRCA in *C9*-ALS: clusters of positive (marked in yellow) and negative (marked in blue) correlation with the cerebellar/brainstem cluster (seed region) are reported on axial sections of a brain Magnetic Resonance Imaging template and on the brain surface of a glass brain rendering (bottom right)

The metabolism of the cerebellar/brainstem cluster was positively correlated with the metabolism of the whole cerebellum, the pons and the medulla oblongata, and negatively correlated with the metabolism of a large cluster including bilateral frontal cortex e left insula (Fig. 3; Table 4).

**Table 4:** IRCA in *C9*-ALS: clusters of positive and negative correlation with the cerebellar/brainstem cluster (seed region). BA: Brodmann area

IRCA in *C9*-ALS: cluster of positive correlation with the cerebellar/brainstem cluster							
*P (FWE- corrected)*	*Cluster extent*	*Z-score*	*Talairach coordinates (x*,* y*,* z)*			*Brain region*	*Sub-region*
0.000	19,759	65,535	14	-69	-25	Right Cerebellar Posterior Lobe	Uvula
		65,535	-30	-53	-38	Left Cerebellar Posterior Lobe	Cerebellar Tonsil
		65,535	16	-68	-37	Right Cerebellar Posterior Lobe	Inferior Semi-Lunar Lobule
		65,535	-10	-70	-28	Left Cerebellar Posterior Lobe	Pyramis
		65,535	-18	-69	-23	Left Cerebellar Posterior Lobe	Uvula
		7.76	-6	-63	-20	Left Cerebellar Posterior Lobe	Declive
		7.71	12	-61	-17	Right Cerebellar Posterior Lobe	Declive
		7.67	-16	-72	-38	Left Cerebellar Posterior Lobe	Inferior Semi-Lunar Lobule
		7.52	26	-47	-40	Right Cerebellar Posterior Lobe	Cerebellar Tonsil
		7.49	-34	-60	-29	Left Cerebellar Posterior Lobe	Tuber
		5.00	-6	-38	-28	Left Cerebellar Anterior Lobe	*
		4.72	8	-36	-27	Right Cerebellar Anterior Lobe	*
IRCA in *C9*-ALS: cluster of negative correlation with the cerebellar/brainstem cluster							
*P (FWE- corrected)*	*Cluster extent*	*Z-score*	*Talairach coordinates (x*,* y*,* z)*			*Brain region*	*Sub-region*
0.000	62,145	65,535	-4	6	33	Left Cingulate Gyrus	BA 24
		7.05	8	39	50	Right Superior Frontal Gyrus	BA 8
		7.00	6	25	28	Right Cingulate Gyrus	BA 32
		6.96	38	46	25	Right Middle Frontal Gyrus	BA 10
		6.95	-32	3	62	Left Middle Frontal Gyrus	BA 6
		6.70	6	9	33	Right Cingulate Gyrus	BA 24
		6.59	30	24	43	Right Middle Frontal Gyrus	BA 8
		6.56	-24	23	36	Left Middle Frontal Gyrus	BA 8
		6.55	-4	34	19	Left Anterior Cingulate	BA 32
		6.48	38	3	61	Right Middle Frontal Gyrus	BA 6
		6.35	63	9	24	Right Inferior Frontal Gyrus	BA 9
		6.35	4	46	20	Right Medial Frontal Gyrus	BA 9
		6.32	-32	49	18	Left Superior Frontal Gyrus	BA 10
		6.32	-2	35	48	Left Superior Frontal Gyrus	BA 8
		6.30	-46	6	3	Left Insula	BA 13

### [^18^F]FDG-PET data: IRCA in ctrl-ALS

**Fig. 4:**
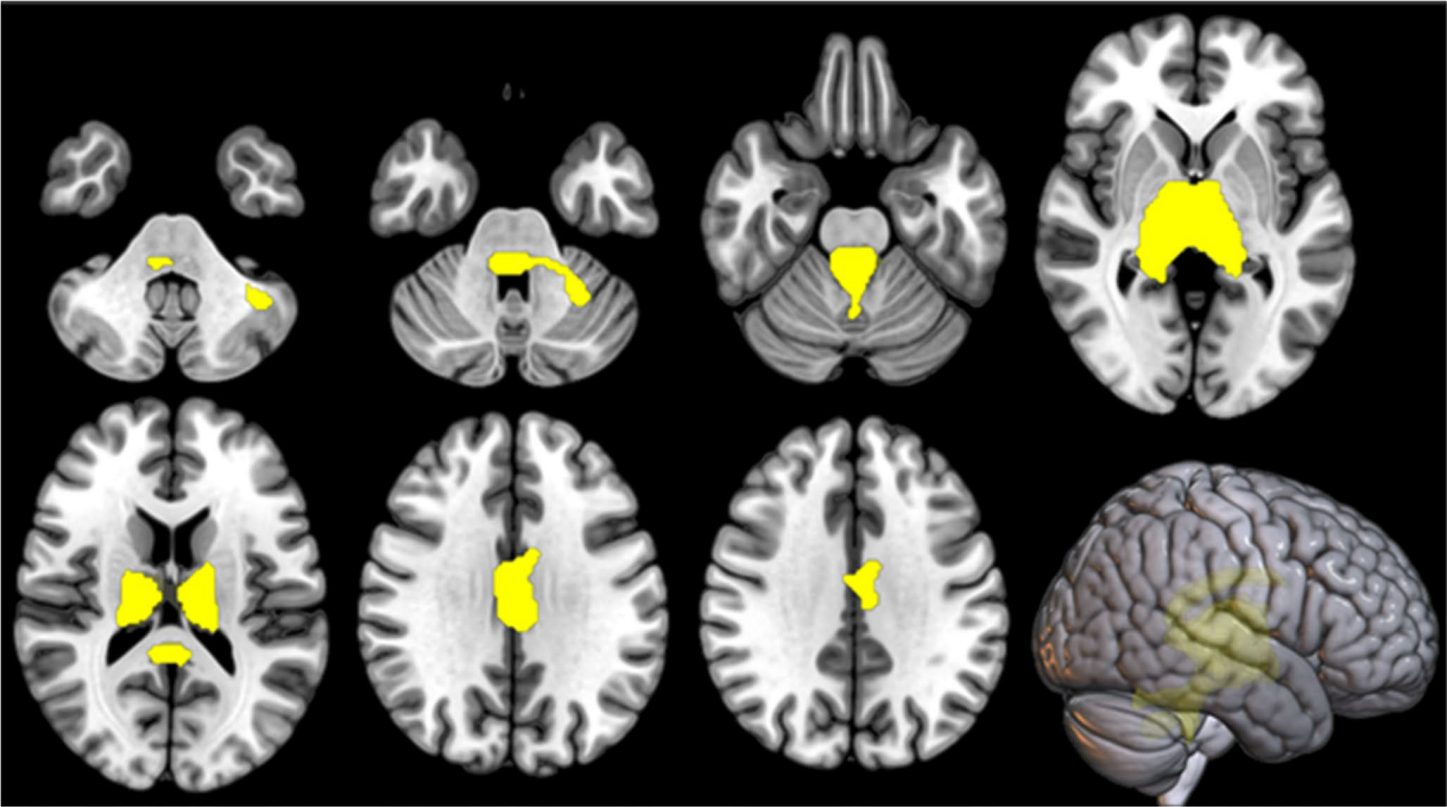
IRCA in ctrl-ALS: the cluster of positive correlation with the thalamic cluster (seed region) is marked in yellow and is reported on axial sections of a brain Magnetic Resonance Imaging template and on the brain surface of a glass brain rendering (bottom right)

In the IRCA in ctrl-ALS subjects, the thalamic cluster showed a positive correlation with a cluster encompassing bilateral cingulate cortex and bilateral cerebellar regions (Fig. 4; Table 5). No negative correlation was found.

**Table 5:** IRCA in ctrl-ALS: cluster of positive correlation with the thalamic cluster (seed region). BA: Brodmann area

IRCA in ctrl-ALS: cluster of positive correlation with the thalamic cluster							
*P (FWE- corrected)*	*Cluster extent*	*Z-score*	*Talairach coordinates (x*,* y*,* z)*			*Brain region*	*Sub-region*
0.000	7,345	65,535	10	-14	1	Right Thalamus	*
		65,535	-8	-23	3	Left Thalamus	*
		65,535	12	-23	1	Right Thalamus	Mammillary Body
		4.40	-2	-24	27	Left Cingulate Gyrus	BA 23
		4.33	-4	-36	22	Left Posterior Cingulate Gyrus	BA 23
		4.13	2	-38	-18	Right Cerebellar Anterior Lobe	Culmen
		3.98	6	-40	17	Right Posterior Cingulate Gyrus	BA 29
		3.64	-40	-54	-29	Left Cerebellar Anterior Lobe	Culmen
		3.37	-18	-38	-23	Left Cerebellar Anterior Lobe	*
		3.18	0	-59	-17	Right Cerebellar Posterior Lobe	Declive

**Fig. 5:**
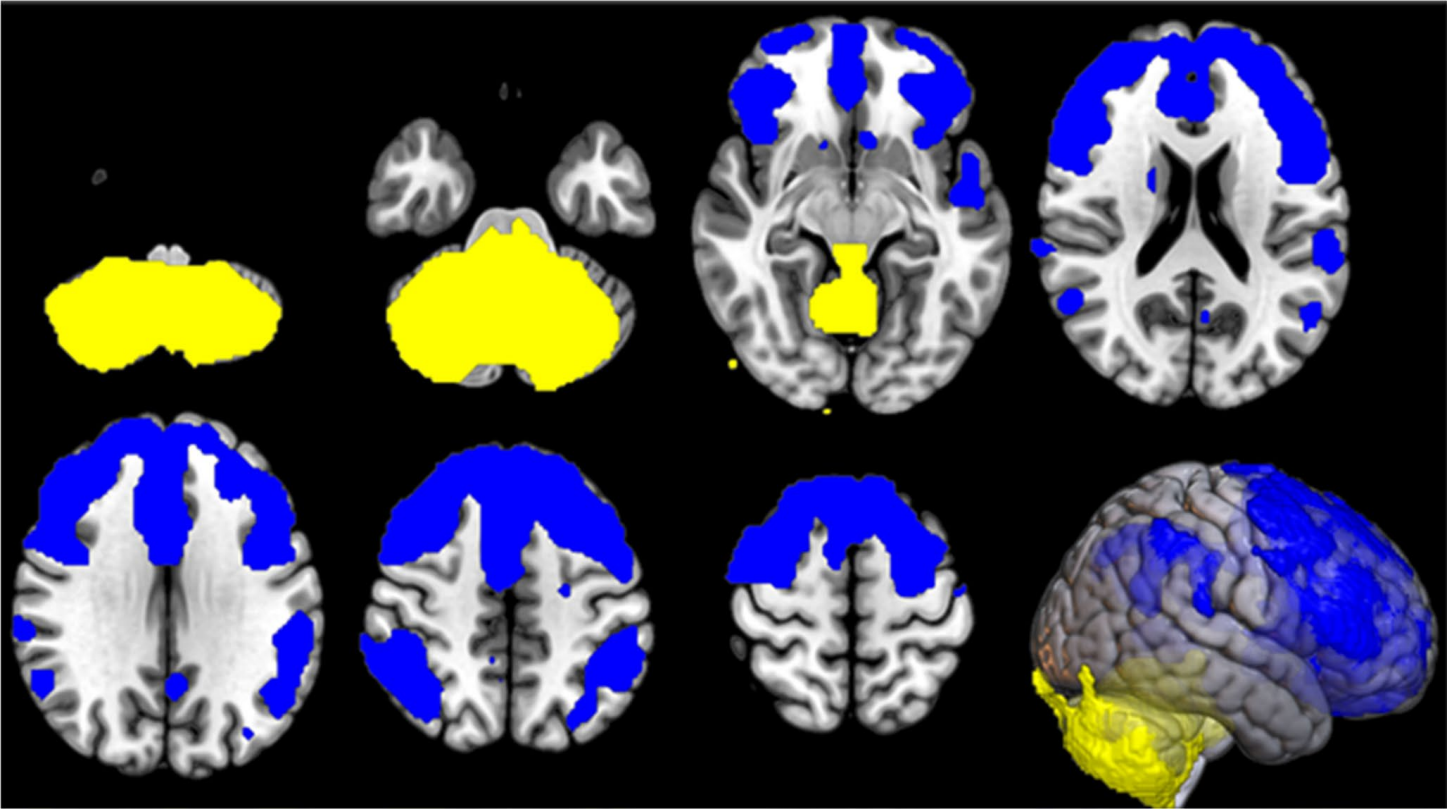
IRCA in ctrl-ALS: clusters of positive (marked in yellow) and negative (marked in blue) correlation with the cerebellar/brainstem cluster (seed region) are reported on axial sections of a brain Magnetic Resonance Imaging template and on the brain surface of a glass brain rendering (bottom right)

The cerebellar/brainstem cluster showed a positive correlation with the whole cerebellum and brainstem and a negative correlation with broad areas encompassing bilateral frontal, temporal and parietal cortices (Fig. 5; Table 6).

**Table 6:** IRCA in ctrl-ALS: clusters of positive and negative correlation with the cerebellar/brainstem cluster (seed region). BA: Brodmann area

IRCA in ctrl-ALS: cluster of positive correlation with the cerebellar/brainstem cluster							
*P (FWE- corrected)*	*Cluster extent*	*Z-score*	*Talairach coordinates (x*,* y*,* z)*			*Brain region*	*Sub-region*
0.000	26,232	65,535	-18	-67	-20	Left Cerebellar Posterior Lobe	Declive
		65,535	12	-69	-22	Right Cerebellar Posterior Lobe	Declive
		65,535	8	-71	-25	Right Cerebellar Posterior Lobe	Pyramis
		65,535	-6	-71	-25	Left Cerebellar Posterior Lobe	Pyramis
		65,535	32	-46	-30	Right Cerebellar Anterior Lobe	*
		65,535	8.0	-51	-18	Right Cerebellar Anterior Lobe	Culmen
		65,535	-28	-54	-24	Left Cerebellar Anterior Lobe	Culmen
		65,535	34	-55	-41	Right Cerebellar Posterior Lobe	Cerebellar Tonsil
		65,535	8	-62	-36	Right Cerebellar Posterior Lobe	Inferior Semi-Lunar Lobule
		7.75	-36	-56	-31	Left Cerebellar Posterior Lobe	Cerebellar Tonsil
		7.75	-24	-44	-26	Left Cerebellar Anterior Lobe	*
IRCA in ctrl-ALS: clusters of negative correlation with the cerebellar/brainstem cluster							
*P (FWE- corrected)*	*Cluster extent*	*Z-score*	*Talairach coordinates (x*,* y*,* z)*			*Brain region*	*Sub-region*
0.000	41,850	6.57	-44	5	24	Left Inferior Frontal Gyrus	BA 9
		6.18	-28	27	39	Left Middle Frontal Gyrus	BA 8
		6.17	-40	32	17	Left Middle Frontal Gyrus	BA 46
		6.12	36	27	41	Right Middle Frontal Gyrus	BA 8
		6.04	8	32	52	Right Superior Frontal Gyrus	BA 6
		6.03	26	43	37	Right Middle Frontal Gyrus	BA 9
		5.78	-4	35	48	Left Superior Frontal Gyrus	BA 8
		5.77	50	12	38	Right Middle Frontal Gyrus	BA 8
		5.77	-30	14	45	Left Middle Frontal Gyrus	BA 6
		5.76	57	31	6	Right Inferior Frontal Gyrus	BA 45
		5.66	50	28	17	Right Inferior Frontal Gyrus	BA 46
		5.62	10	47	40	Right Superior Frontal Gyrus	BA 8
		5.60	28	12	55	Right Middle Frontal Gyrus	BA 6
		5.45	12	34	19	Right Anterior Cingulate	BA 32
		5.42	34	55	14	Right Superior Frontal Gyrus	BA 10
0.000	3,820	5.30	-51	-46	47	Left Inferior Parietal Lobule	BA 40
		4.27	-51	-59	29	Left Superior Temporal Gyrus	BA 39
		3.89	-55	-48	8	Left Superior Temporal Gyrus	BA 22
		3.79	-53	-56	6	Left Middle Temporal Gyrus	BA 39
		3.77	-30	-70	42	Left Precuneus	BA 19
		3.73	-63	-42	6	Left Middle Temporal Gyrus	BA 22
0.000	2,376	4.54	53	-51	25	Right Supramarginal Gyrus	BA 40
		4.54	53	-44	46	Right Inferior Parietal Lobule	BA 40
		4.08	38	-58	47	Right Superior Parietal Lobule	BA 7
		3.76	63	-25	34	Right Postcentral Gyrus	BA 2
		3.71	69	-30	20	Right Superior Temporal Gyrus	BA 42

## Discussion

The purpose of this study was to investigate the brain metabolic connectivity in ALS patients with *C9ORF72* expansion. To this end, we analysed a sample of 70 patients with *C9ORF72* mutation (*C9*-ALS) and 70 ALS patients without genetic mutations serving as the reference group (ctrl-ALS). The *C9*-ALS group was representative of the subgroup of patients carrying such mutation in our population-based register (PARALS), except for a lower degree of FTD in our sample. Despite this difference, we considered the group suitable for our purpose, accepting the risk of underestimating the differences compared with the ctrl-ALS group. The ctrl-ALS group was fully representative of the PARALS population.

In a direct comparison between *C9*-ALS and ctrl-ALS, [^18^F]FDG-PET imaging revealed relative hypometabolism in the thalami - specifically the bilateral pulvinar and left medial dorsal nucleus - and in the left precentral and postcentral gyri in *C9*-ALS. These findings confirm and extend previous MRI studies reporting reduced cortical thickness and gray matter volume in the same regions [18], supporting the notion that [^18^F]FDG-PET hypometabolism and structural atrophy are complementary indicators of neurodegeneration [19].

Thalamic changes, particularly hypometabolism and volumetric reduction, have been consistently reported in both symptomatic [8, 20] and presymptomatic *C9ORF72* expansion carriers [21, 22]. Pathologically, the thalami in these individuals are known to accumulate both TDP-43 [17] and dipeptide repeat (DPR) proteins [23, 24], the latter produced through repeat-associated non-ATG (RAN) translation of the hexanucleotide expansion. Thalamic damage appears to be part of a broader network degeneration associated with psychotic symptoms, commonly seen in *C9ORF72* patients [25], similar to networks implicated in schizophrenia [26].

A prior study on behavioural variant frontotemporal dementia (bvFTD) [27], often associated with ALS [28], showed that about one-third of bvFTD patients - mostly *C9ORF72* carriers - had predominant subcortical atrophy and slower disease progression. This may explain the predominantly subcortical hypometabolism in our *C9*-ALS sample, as more cognitively impaired patients may have been underrepresented due to lower compliance with PET protocols. This is also consistent with the lower proportion of full-blown FTD in our *C9*-ALS group compared to the broader population of *C9ORF72* expansion carriers in our epidemiological register (PARALS).

Focusing on the thalamic hypometabolic cluster, we used IRCA to assess metabolic connectivity. The thalamic seed region showed a broader positive correlation with the cingulate cortex, particularly its anterior region, in *C9*-ALS than in ctrl-ALS, suggesting a stronger involvement of the Salience Network (SN). The SN, which includes the pulvinar, medial dorsal thalamic nucleus, and anterior cingulate cortex, is crucial for processing emotionally relevant stimuli and supporting behavioural and cognitive control [20, 29]. Functional MRI data also support the idea that pulvinar degeneration contributes to bvFTD symptoms in *C9ORF72* carriers through SN disruption [30]. The SN’s connectivity might also act as a path for spreading neurodegeneration, as proposed in other studies on neurodegenerative disorders [31].

Conversely, a negative correlation between the thalamic seed and the sensorimotor cortex was observed in *C9*-ALS but not in ctrl-ALS. Interestingly, this pattern resembles thalamic hyperconnectivity with sensorimotor cortex found in schizophrenia, even when thalamic atrophy is present, suggesting possible shared network dysfunction mechanisms [32].

Additionally, *C9*-ALS patients exhibited relative hypermetabolism in the brainstem and cerebellum compared to ctrl-ALS. These hypermetabolic patterns have been observed in ALS patients with cognitive impairment [33, 34]. Although cognitive status did not differ significantly between the two groups, there was a higher proportion of FTD cases in the *C9*-ALS group, supporting the relevance of these metabolic findings, as [^18^F]FDG-PET can reveal brain metabolic changes even in the absence of overt symptoms [21].

From a pathological perspective, TDP-43 proteinopathy rarely involves the cerebellum in ALS except in late stages, while the brainstem is affected early [35]. However, in *C9ORF72* expansion carriers, the cerebellum is often affected by ubiquitin- and p62-positive, DPR-containing neuronal cytoplasmic inclusions [17]. These data demonstrate the involvement of the cerebellum in *C9ORF72* expansion carriers, but are also compatible with its relative sparing compared to the frontal regions, as neurodegeneration appears to be more strongly associated with TDP-43 proteinopathy than with DPR [36].

To further explore cerebellar and brainstem involvement in *C9*-ALS and ctrl-ALS, we conducted IRCA using these regions as seeds in both groups. While positive correlations largely reflected local autocorrelation, negative correlations with frontal cortices were more extensive in *C9*-ALS. This inverse relationship between cerebellar and frontal metabolism may reflect a compensatory mechanism, with cerebellar regions attempting to offset declining frontal lobe function. This hypothesis seems to be supported by a previous study about cognitive reserve in ALS [37] and by neuroimaging and neuromodulation/neurostimulation studies suggesting that the cerebellum may undergo a compensatory reorganisation in neurodegenerative disorders causing cognitive impairment, such as AD and FTD [38].

However, this compensatory process might diminish over time, as the cerebellum becomes affected by TDP-43 pathology in advanced ALS stages [35], particularly in *C9ORF72* carriers [17]. Thus, both degenerative and compensatory mechanisms may coexist in the cerebellum of ALS patients [39]. This complex interplay likely contributes to the metabolic and connectivity patterns observed in our study.

In summary, our findings suggest that *C9*-ALS is characterised by specific subcortical and network-level alterations, including thalamic and cerebellar metabolic changes, which may reflect both disease-specific neurodegeneration and potential compensatory responses. These insights deepen our understanding of how brain metabolic connectivity differs in genetically distinct ALS subtypes and underscore the role of intrinsic brain networks in ALS pathophysiology.

This study has some limitations. Firstly, our *C9*-ALS sample showed a lower percentage of cases with cognitive impairment up to FTD than *C9ORF72* expansion carriers in our population-based register. This may have limited our findings, possibly due to reduced compliance with PET acquisition among FTD subjects, although it is difficult to fully assess this issue. Secondly, exhaustive and systematic data about psychotic symptoms are not available for the two study groups, thus we could not evaluate their relationship with thalamic metabolic changes, that seem a distinctive feature of ALS with *C9ORF72* expansion. Regarding the possibility of identifying subgroups according to motor phenotypes, it would not provide adequately sized samples to run further analyses. Thirdly, Structural MRI scans were not available for all subjects, so we could not correct for cortical atrophy. Nevertheless, previous studies have shown that measures of metabolism were relatively independent of brain atrophy [40].

In conclusion, our study shows that *C9*-ALS patients have a relatively lower metabolism mainly in the thalami and a relatively higher metabolism in the brainstem and the cerebellum compared to ctrl-ALS. Together with the analysis of the metabolic connectivity of these regions, these data suggest a twofold hypothesis. On the one hand, *C9*-ALS seem to show a predominant involvement of the SN compared to ctrl-ALS. The SN is related to cognitive and behavioural control and could act as a pathway for brain degeneration. On the other hand, the cerebellum may be recruited to cope with cognitive impairment to a greater extent in *C9*-ALS than in ctrl-ALS. Further studies including longitudinal assessments are warranted to further elucidate *C9ORF72*-related changes in brain integrity and connectivity.

## Data Availability

The data that support the findings of this study are available on request from the corresponding author. The data are not publicly available due to privacy or ethical restrictions. [Edit]
